# Dairy foods and osteoporosis: an example of assessing the health-economic impact of food products

**DOI:** 10.1007/s00198-012-1998-6

**Published:** 2012-06-16

**Authors:** F. J. B. Lötters, I. Lenoir-Wijnkoop, P. Fardellone, R. Rizzoli, E. Rocher, M. J. Poley

**Affiliations:** 1 Institute of Health Policy and Management (iBMG), Erasmus University Rotterdam, Rotterdam, The Netherlands; 2 Departement of Pharmaceutical Sciences, University of Utrecht, Sorbonnelaan 16, 3508TB Utrecht, The Netherlands; 3 Danone Research, Route Départementale 128, 91767 Palaiseau, France; 4 Service de Rhumatologie, Centre Hospitalier Universitaire Amiens, INSERM ERI 12, Amiens, France; 5 University Hospital Geneva, Bone Diseases, Geneva, Switzerland; 6 Institute for Medical Technology Assessment (iMTA), Erasmus University Rotterdam, Rotterdam, The Netherlands

**Keywords:** Calcium, Dairy products, Hip fractures, Nutrition economics, Osteoporosis

## Abstract

**Summary:**

Osteoporosis has become a major health concern, carrying a substantial burden in terms of health outcomes and costs. We constructed a model to quantify the potential effect of an additional intake of calcium from dairy foods on the risk of osteoporotic fracture, taking a health economics perspective.

**Introduction:**

This study seeks, first, to estimate the impact of an increased dairy consumption on reducing the burden of osteoporosis in terms of health outcomes and costs, and, second, to contribute to a generic methodology for assessing the health-economic outcomes of food products.

**Methods:**

We constructed a model that generated the number of hip fractures that potentially can be prevented with dairy foods intakes, and then calculated costs avoided, considering the healthcare costs of hip fractures and the costs of additional dairy foods, as well as the number of disability-adjusted life years (DALYs) lost due to hip fractures associated with low nutritional calcium intake. Separate analyses were done for The Netherlands, France, and Sweden, three countries with different levels of dairy products consumption.

**Results:**

The number of hip fractures that may potentially be prevented each year with additional dairy products was highest in France (2,023), followed by Sweden (455) and The Netherlands (132). The yearly number of DALYs lost was 6,263 for France, 1,246 for Sweden, and 374 for The Netherlands. The corresponding total costs that might potentially be avoided are about 129 million, 34 million, and 6 million Euros, in these countries, respectively.

**Conclusions:**

This study quantified the potential nutrition economic impact of increased dairy consumption on osteoporotic fractures, building connections between the fields of nutrition and health economics. Future research should further collect longitudinal population data for documenting the net benefits of increasing dairy consumption on bone health and on the related utilization of healthcare resources.

## Introduction

Health benefits of dairy foods, which provide a large variety of essential nutrients such as minerals, vitamins, and proteins, are widely recognized [1]. Dairy foods, consumed by many people throughout the Western world as part of the daily diet [2, 3], are a determinant of human health and well-being. Although the extent of those effects has not been completely unfold, some of the reported benefits concern the area of cardiovascular diseases, colorectal cancer, obesity and type 2 diabetes [4–6]. Several studies have documented the link between the intake of dairy foods and osteoporosis, associating low dietary calcium intake with decreased bone density and osteoporotic fractures, as dairy products consistently provide 60 % to 70 % of daily calcium intakes [7–12]. In a review by McCarron and Heaney on the effects of dairy products in several medical conditions, they concluded that in the USA intake of the recommended quantities of dairy products would yield 5-year savings (limited to healthcare costs) of $209 billion. Of this, $14 billion relate to savings on the healthcare costs for osteoporosis (limited to treating fractures) [13]. Over the past decades, osteoporosis has become a major health concern, estimated to affect over 200 million people worldwide [14, 15]. The disease carries a substantial burden. First, osteoporosis increases the risk of fractures, associated with increased mortality, increased morbidity, limitations in physical function, pain, and losses in health-related quality of life [16, 17]. Second, osteoporotic fractures considerably increase healthcare costs, in both inpatient and outpatient settings, as has been confirmed by several studies [18–20]

Calcium and vitamin D supplementation, anti-osteoporotic drugs, and exercise programs have been shown to be effective in reducing the risk of fractures [21, 22]. However, in daily practice non-compliance appears to be a significant problem with specific anti-osteoporotic therapy and with calcium and vitamin D supplementation as well [23, 24]. This provides a rationale for supporting a more food-oriented preventive approach of osteoporosis.

The purpose of this study was to explore the relationship between a food-related health condition and its potential impact on health care expenditures. Currently, the literature contains hardly any relevant studies on the impact of dairy foods on healthcare costs or cost-effectiveness [25, 26]. Despite the fact that the effects of foods on health are increasingly recognized, there is no accepted, proven methodology to assess the health-economic impact of foods in the general population. The scarcity of estimations on the health-economic impact of foods stands in sharp contrast with the ever-growing evidence on the cost-effectiveness of (public) health technologies [27, 28]. Obviously, the evidence most adapted to a general population setting as well to the long latency periods for nutrition-related diseases mainly has to come from prospective cohort studies with disease events and death as outcome.

In this paper, we propose an approach for estimating the potential nutrition economic impact of dairy products on the burden of osteoporosis in the general population over 50 years of age. The aims are first, to quantify the burden of osteoporosis (in terms of costs and health outcomes) and to estimate the potential impact of increasing dairy foods consumption on reducing this burden. These calculations were performed for France, The Netherlands, and Sweden. Secondly, this study aims to contribute to the development of a generic methodology for assessing the health-economic outcomes of food products.

## Materials and methods

### Data sources

Systematic literature reviews were performed using the following sources: PubMed library, Cochrane library, Embase, and Scopus; Health-economic databases, such as EURONHEED, the NHS Economic Evaluation Database (NHS EED), and the CEA Registry maintained by the Center for the Evaluation of Value and Risk in Health.

We used the following search criteria: major search terms were osteoporosis, fracture, bone, dairy, milk, calcium, vitamin D, intervention, supplementation, mortality, quality of life, QALY, medical consumption, costs, cost-effective, cost-benefit, and economic evaluation; peer-reviewed articles were included; only articles in English or Dutch were taken into account; the site of the fracture (at least studies had to distinguish hip fractures from other fractures like spine and wrist); review studies that did not include new data were excluded; studies published in 1995 and onwards were included; due to insufficient data available in the literature on all age groups, the search was restricted to age 50 years and older.

The studies retrieved by the literature search were used to arrive at valid estimations of the following parameters, which were needed as an input to the model:

- Relationship between calcium intake by dairy foods and osteoporotic fractures indicated by relative risk estimates or odds ratios.
- Costs of treating fractures in the first year and in subsequent years.
- Mortality risk associated with osteoporotic fractures.
- Health-related quality of life (‘utilities’) of healthy people and of people who are experiencing an osteoporotic fracture; studies had to use generic (not disease specific), preference based instrument to come to a utility.

The way how the above mentioned parameters were retrieved or calculated in each study was critically judged. Studies that divided their results in age classes were preferred. Studies that evaluated the effects of a specific treatment modality (in a subgroup of patients), rather than including a ‘broad’ population sample of patients with fractures, were excluded.

We derived data from national databases for each country, i.e. Statistics Netherlands (CBS; www.cbs.nl), National Institute of Statistics and Economic Studies (INSEE; www.insee.fr), and Statistics Sweden (SCB; www.scb.se). For The Netherlands, we also used results of the Dutch National Food Consumption Survey [29].

**Fig. 1:**
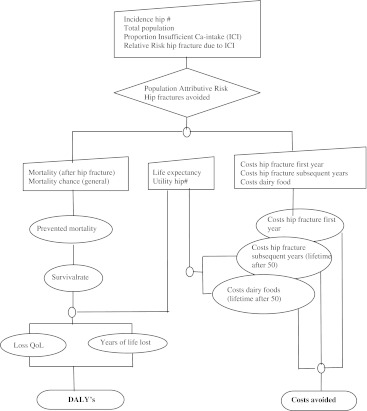
Flow diagram of the nutrition-economic model for hip fracture as outcome of osteoporosis

The data needed to build our nutrition economic model can be found in the flow diagram presented in Fig. 1.

### Study population and countries

The populations of interest were men and women (of any ethnicity) from the general population of Western Europe aged 50 years and over. This includes individuals treated and not treated for osteoporosis. We specifically looked for data that divided the (general) population by sex and 5-year age classes.

Health-economic studies should take into account differences between countries. In this case, it can be expected that dairy intakes may differ considerably between different regions in Europe [3]. Moreover, other differences between the populations of several countries may affect the occurrence of osteoporosis, such as lifestyle, the availability and quality of healthcare, climate, genetic predisposition, etc. Furthermore, treatment pathways, costs of healthcare, and cost prices of dairy food products will differ. To get insight in country differences we will present the model outcomes for The Netherlands, Sweden, and France, a choice based on different dairy intakes and on the availability of country specific public health data and nutritional surveys.

### Study focus and perspective

Because dairy food products are the major source of calcium in the Western European diet, this study aimed at quantifying the potential impact of increasing dairy foods consumption on the occurrence of osteoporotic hip fractures in people with an inadequate calcium intake. We defined low calcium intake as a daily intake equal to or less than 600 mg, which is approximately half of the daily intake (DRI) recommended by the International Osteoporosis Foundation [30, 31]. We used the calcium content of dairy foods as a marker to model the effect on osteoporotic hip fractures.

The study primarily analysed the costs and health impact from a healthcare perspective. In addition to this, we broadened the perspective to a more societal approach by including the costs of dairy foods made by those persons who could be prevented from having a hip fracture associated with low calcium intake. The study took a life-long time horizon, which implies that both costs and effects were taken into account from the occurrence of hip fracture till death. We used the discount rates recommended in the Dutch guidelines for pharmaco-economic research (that is, 4 % for costs and 1.5 % for effects) [32].

### Analytical techniques and main outcome measures

Using the risk estimate found in the literature, we calculated the Population Attributive Fraction (PAF). This represents the percentage of all hip fractures (among exposed and unexposed) that can be attributed to low calcium intake, as expressed in the formula:

$$ {\text{PAF}} = \left[ {{{\text{P}}_{\text{e}}}\left( {{\text{RR}} - {1}} \right)} \right]/\left[ {{{\text{P}}_{\text{e}}}\left( {{\text{RR}} - {1}} \right) + {1}} \right] $$

where: P_e_ = prevalence of risk factor in the population; RR = relative risk for hip fracture due to low calcium intake [33].

Next, we calculated the absolute amount of hip fractures that potentially can be prevented with additional calcium intake. In epidemiology, this number is known as the ‘potential impact fraction’ (PIF), i.e. the potential reduction in disease prevalence resulting from a risk factor intervention program. It is calculated by multiplying (per age class) the incidence of hip fractures with the corresponding PAF for that age class [33]. In a formula:

$$ {\text{PIF}} = {\text{I}}\;*\;{\text{N}}/{1},000\;*\;{\text{PAF}} $$

where: I = incidence of hip fractures (per 1,000); N = total population per age class; PAF = population attributive fraction. This measure will be used in the further calculations in the model, i.e. the outcomes disability-adjusted life years (DALYs) and costs avoided will be referring to the total population per age class.

In order to assess the potential impact of increased dairy consumption on the prevention of osteoporotic hip fractures, our model includes two main outcome measures. The first is costs avoided. These are calculated by determining the costs of treating hip fractures (i.e. healthcare costs made in the first year after a fracture, as well as those made in subsequent years) and subsequently subtracting the costs made for extra dairy food consumption. In the primary analyses, only the costs of dairy food products made by those people in whom a hip fracture actually could be avoided by extra dairy food consumption are taken into account: we calculated how many dairy products people with a low calcium intake would have to consume to reach the DRI. The data on the calcium content of dairy products were taken from the Dutch Food Composition Database (NEVO) [34]. We took an average of different types of dairy products—including milk, yogurt, fresh cheese, and cheese—representing the common consumption pattern in the population for each of the three countries. For example in The Netherlands, extra 650 mg calcium per day equaled: 200 milliliter low-fat milk (=242 mg calcium) + 125 milliliter low-fat yogurt (=166 mg calcium) + 30 gram young cheese (=237 mg calcium). These data were combined with country-specific unit cost prices of dairy products, derived from general market prices (September 2010 prices). To facilitate comparisons, we used the prices of national supermarkets (preferably the market leaders) rather than those of traditional shops. Finally, we arrived at total costs per day/year, representing the total additional costs if people with a low calcium intake raise their intake up to the recommended level by increasing their dairy foods consumption.

The second main outcome of our model is the number of lost DALYs, which represent a widely-used summary indicator of public health [35]. DALYs are the sum of life years lost due to premature mortality and years lived with disability adjusted for severity. In other words, the basic formula for DALYs is:

$$ {\text{DALY}} = {\text{YLL}} + {\text{YLD}} $$

where: YLL = years of life lost due to premature mortality; YLD = years of healthy life lost as a result of disability.

The DALY measure was used to calculate the life years lost and the loss in quality of life due to hip fracture caused by low calcium intake (see Fig. 1). We used country- and age-specific mortality rates due to hip fracture. In this respect, it is important to distinguish between excess mortality rates, i.e. the proportion of the population suffering from a hip fracture that dies, and general population mortality, i.e. the proportion of the general population that dies due to hip fracture [36]. Considering the data available, and for reasons of comparability between countries, we used the mortality rates after hip fracture in the general population.

### Sensitivity analyses

We conducted sensitivity analyses to verify to what extent certain assumptions might have influenced the results. Plausible ranges of uncertain parameters were obtained from the published literature or by varying the estimates by a certain percentage in each direction. The following parameters were varied:

1. The relative risk expressing the relationship between a low calcium intake and the occurrence of hip fractures, and the proportion of the general population with a low calcium intake.
2. Long-term quality of life impact of hip fractures.
3. Discount rates.
4. Dairy food costs.

The results of the sensitivity analyses are expressed in the outcome measures of DALYs lost and total costs avoided.

## Results

Table 1 shows the data used as input in the model. For the sake of clarity, the table pools the data from both sexes and all age categories. In the model itself, all input variables were divided into sex and age categories (i.e. 50–54, 55–59, 60–64, 65–69, 70–74, 75–79, 80–84, ≥85 years). The risk factor for a hip fracture due to low calcium intake was based on a study by Cumming et al., and amounted to 1.08 [37]. The incidence of hip fractures in both men and women in Sweden appeared to exceed that of The Netherlands and France. Moreover, in all countries, it shows that the incidence of hip fractures in women is higher compared with men. Furthermore, the incidence of hip fractures and mortality rates after hip fracture increase substantially with age especially in the age categories of 70 and above. As explained above, the mortality figures in Table 1 refer to the mortality after hip fracture in the general population. It appeared that, up to the age of 80 years, the mortality data for Sweden exceed those for The Netherlands and France, probably because of the high incidence rates of hip fracture in Sweden compared to the other countries. In the first year after hip fracture, the average loss of quality of life (‘utility’) was calculated at 0.22; while in the following years, the average loss of quality of life was 0.08. Daily costs of additional dairy products were calculated at € 0.44, € 0.64, and € 0.68, for The Netherlands, France, and Sweden, respectively.

**Table 1:** Summary of data used and its sources (all age categories pooled)

Parameter	Data (mean over both sexes) (>50 years)			Data sources
	NL	FR	SE	NL/FR/SE
Percentage of low calcium intake (i.e., <600 mg/day) in the general population	8 %	40 %	31 %	[11, 43, 69]
Recommended intake of calcium in the elderly (mg/day)	1,300	1,300	1,300	[30]
Incidence of hip fractures (per 1,000)^f^	53.9	35.2	64.7	RIVM^a^ [36, 70]
Size of the general population (absolute numbers)^f^	5,603,463	21,689,920	3,378,795	CBS^b^/INSEE^c^/SCB^d^
Relationship between a low calcium intake and hip fractures: RR (95 % CI)	1.08 (1.02-1.16)	1.08 (1.02-1.16)	1.08 (1.02-1.16)	[37]
Costs of hip fractures (in Euro)^f^				[59, 71, 72]
-First year after the fracture	€ 129,210	€ 114,602	€ 114,025	
-Subsequent years	€ 22,815	€ 50,488	€ 50,700	
General mortality following hip fractures (per 10,000)	28.7	35.9	99.5	CBS [36, 73]
Life-expectancy (years) and mortality (chance) in the general population (at 50 years)	28.9	30.5	30.6	CBS/INSEE/SCB
	0.038	0.033	0.033	
Health-related quality of life following hip fractures (i.e., the reduction in quality of life measured on a scale from 0 to 1)				[38]
-First year after the fracture	0.22	0.22	0.22	
-Subsequent years	0.08	0.08	0.08	
Unit cost prices of dairy foods; ‘intervention costs/ day’ (in Euro)^e^	€ 0.44	€ 0.64	€ 0.68	Albert Heijn (www.albert.nl)
				Carrefour (www.carrefour.fr)
				ICA (www.ica.se)

*CBS* Statistics Netherlands, *INSEE* Statistics France, *IOF* International Osteoporosis Foundation, *SCB* Statistics Sweden

^a^ http://www.nationaalkompas.nl

^b^ http://www.cbs.nl

^c^ http://www.inseee.fr

^d^ http://www.scb.se

^e^Corresponding to an extra 650 mg calcium per day; September 2010 prices

^f^Summed over the eight distinguished age categories

### Main outcomes

**Fig. 2:**
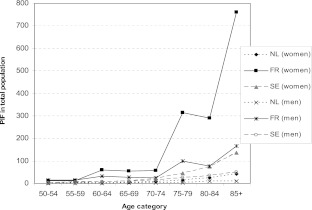
Potential impact fraction (absolute numbers)

With a distinction according to age class, Fig. 2 shows the PIF, indicating the number of hip fractures that could potentially be prevented each year with additional calcium intake. All age classes taken together, the PIF is highest in French women (1,565), followed by Swedish women (307). Across all age classes, the PIF number was relatively low in The Netherlands (103), compared with France and Sweden.

The prevented mortality is relatively low for all three countries: all age classes and both sexes taken together, the number of deaths prevented per 10,000 persons experiencing a hip fracture is 5.1 (Sweden), 2.4 (France), and 0.4 (The Netherlands), respectively. This can be explained by the fact that the PAF (i.e. the percentage of hip fractures attributed to low calcium intake) is rather low (The Netherlands, 0.8 %; France, 3.1 %; and Sweden, 2.2 %).

**Fig. 3:**
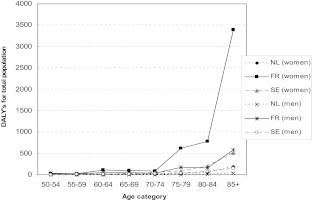
DALYs lost, representing the burden of hip fractures in relation to low calcium intake

Figure 3 shows the yearly number of DALYs lost, representing the burden of hip fractures due to low calcium intake. In all countries, the number of DALYs lost appears to increase with age. In total, the yearly societal burden of hip fractures due to low calcium intake appeared to be 6,263 DALYs for France, 1,246 DALYs for Sweden, and 374 DALYs for The Netherlands.

**Fig. 4:**
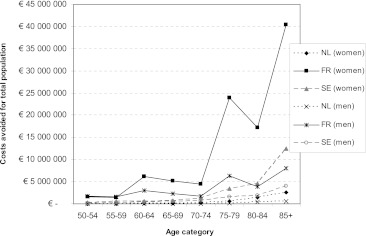
Costs avoided (first and subsequent years after hip fracture) through improved dairy foods consumption

Figure 4 shows the total costs that can potentially be avoided when the risk of hip fractures is decreased by the additional consumption of dairy foods. These discounted costs (which are actually savings) represent the difference between the costs of treating hip fractures and the costs of extra dairy foods. The potential savings on the costs of treating hip fractures exceeded the costs of extra dairy foods in all age classes in all three countries. The total costs potentially avoided were largest in women in France (€ 100,311,274) followed by women in Sweden (€ 23,912,460) and The Netherlands (€ 5,121,041). The main part of these costs can be prevented in the older age categories, i.e. from 70 years onwards.

### Sensitivity analyses

We varied the PAF by changing the risk factor for a hip fracture associated with low calcium intake (using the 95 % confidence interval of 1.02 to 1.16) [37], as well as by changing the proportion of people with a low calcium intake. Both outcomes of the model (i.e. number of DALYs lost and costs avoided) are sensitive for the relative risk of a hip fracture, from age category 70–74 onwards. For example, in the case of The Netherlands, the number of DALYs lost in women aged 85 years and above (in the primary analysis calculated at 185) ranged from 46 to 367. In this subgroup, varying the relative risk made the costs avoided fluctuate between € 0.6 million and € 5.1 million (in the primary analysis calculated at € 2.6 million). When changing the proportion of people with a low calcium intake with 10 %, the number of DALYs and the costs avoided will concomitantly change with approximately 10 %.

The quality of life after hip fracture during subsequent years was changed using a range of 0.05 and 0.12, where 0.08 was used in the primary analyses [38]. This did not substantially change the outcomes for the three countries under study.

In the primary analyses, a discount rate of 4 % for costs and 1.5 % for health effects was used. We compared this to the results without discounting. The analysis showed that both outcomes (DALYs and costs avoided) were, as expected, slightly lower than when discounting is applied.

Finally, a calculation of costs avoided was made in case dairy food costs were omitted from the model. The reason to do so is that the extra dairy food consumption will most likely be a substitute for other food products. This analysis revealed slightly higher costs savings (3 %).

## Discussion

In this study, we quantified the potential nutrition economic impact of increasing dairy consumption by people with low calcium intake on the occurrence of osteoporotic hip fractures. The core of the model was the absolute amount of hip fractures that potentially can be prevented. We particularly paid attention to the potential preventive effect of increasing calcium intake on the occurrence of hip fractures, DALYs, and costs in the population at risk. By including several, geographically distinct European countries with different food patterns, it was shown how the nutrition economic impact of dairy foods on hip fractures varies between countries with different incidence rates of hip fractures, different numbers of people with low calcium intake, and different costs of healthcare and costs of dairy foods. Our study concentrated on middle-aged and older groups, aged 50 years and over.

One may question to which extent the principles of health economics apply to food products and dietary habits. Will it simply come down to applying the principles and methods of health economics, or would it be required to develop ‘nutrition economics’, as a novel subarea of health economics [25]? Next to similarities between health economics in general and ‘nutrition economics’ in particular, there also will be differences, for example relating to differences in study populations and relating to the fact that food-related changes are often relatively small and only observable over a long time window [39, 40].

There is a need to work towards a generic methodology to assess the impact of foods on health, well-being, and costs. In making this effort, osteoporosis offers an excellent case study: it represents a heavy burden and has a high prevalence, the disease is progressing slowly and has an early onset (several decades before it actually manifests itself), and is associated with food consumption [9].

In accordance with earlier studies [41], the incidence of hip fractures was highest for Sweden, compared to The Netherlands and France. One explanation for these inter-country differences may be related to different levels of calcium intake between countries’ populations. However, there will be other explanations as well, which is why there is no one-to-one relationship between calcium intake and rates of hip fractures (as the numbers for the countries included in this study demonstrate). Plausible other hypotheses for these inter-country differences include genetic predisposition and lifestyle factors (nutritional patterns in general, physical activity, etcetera) [42]. The highest PIF was found in French women, which can be explained by the relatively large proportion of the French female population with a low calcium intake. In The Netherlands, this PIF number was much lower, relating to the fact that the Dutch consume large amounts of dairy foods [43, 44]. It should be noted that the food consumption studies used measured calcium intake from all food products, not solely dairy foods. However, dairy foods contributed by far the most to calcium intake [11, 43].

The yearly societal burden of hip fractures associated with low calcium intake appeared to be 374 DALYs for The Netherlands, 6,263 DALYs for France, and 1,246 DALYs for Sweden. The potential savings on the costs of treating hip fractures exceeded the costs of extra dairy foods in all three countries. Total costs avoided were largest in France, mainly due to the relatively high PIF found in France. As mentioned before, the main calculations rested on the assumptions that all these hip fractures are indeed prevented. This might raise questions about compliance. It is known that compliance with current anti-osteoporotic drugs is rather low, and optimal anti-fracture efficacy is not always achieved in clinical practice [23, 45, 46]. In a recent study [47], dairy food has been shown to be an appropriate vehicle to supplement extra calcium and other minerals, with good compliance compared to that reported for supplements [48].

The daily costs of additional dairy were lowest in The Netherlands, compared to France and Sweden. This corresponds with the findings of a European Commission report, which analysed price differences of supermarket goods across Europe [49]. In the primary analysis, costs of additional dairy foods were applied only to those persons who actually could be prevented from having a hip fracture due to low calcium intake. This might overestimate the outcome of the model, as, from a primary prevention point of view, one needs to expose the whole population at risk to extra calcium intake by means of extra dairy consumption. It might be assumed though that when the people at risk start taking extra dairy, this will be a substitution—either full or partly—for other food products. Hence, in this situation, the total cost of dairy foods might only be slightly higher. If a strict health care perspective is adopted, the costs of purchasing dairy foods as part of a normal diet do not need to be taken into account. The scope of the analysis can be limited to the health care costs made for hip fractures.

Some remarks should be made on the data used as input in the calculations, especially regarding the relative risk for hip fracture associated with low calcium intake. First, reviews with pooled study results do not take into account different starting levels of calcium intake. This might hamper the interpretation of the effect size of low calcium intake on the occurrence of hip fractures. The data existing in the literature did not allow us to correct for a different start point in calcium intake of these elderly in our model. This probably resulted in an underestimation of the effect size of the main outcomes in this study. Second, the relative risk for hip fracture was derived from the meta-analysis of Cumming et al. [37]. Although more recent studies are available on the relationship between calcium intake and osteoporotic fracture, this study mentioned a dose–response relationship. In another meta-analysis, it was found that a supplement of 500 to 1,200 mg calcium would reduce the risk of hip fracture with 12 % (RR 0.88; 95 % CI 0.83–0.95) [50]. This study only took into account randomized controlled trials, with calcium supplementation as intervention. However, both studies are concordant. Recently, a meta-analysis by Bischoff-Ferrari et al. did not find a significant reduction in hip fracture by drinking milk for men and women [51]. However, by deleting a Swedish study (considered to be an outlier) from their analyses, the authors found a statistically significant risk reduction of 5 %. Also in a meta-analysis by Kanis et al. [44], it was found that a low intake of milk was not associated with a marked increase in hip fracture risk. However, low intake was defined as drinking less than one glass of milk daily. Dairy products such as cheese and yogurt were not taken into account. We defined low calcium intake to be under 600 mg, we took a risk reduction of 8 % based on the data of Cumming et al [37], thereby following a conservative approach. Finally, our approach was supported by the results of a recent population-based cohort study by Warensjö et al. In this study, it was found that a dietary calcium intake below approximately 700 mg per day in women was associated with an increased risk of hip fracture [52]. This risk estimate was somewhat higher than in our study. However, this comprehensive study was not specifically directed at dairy calcium intake.

We only used low calcium intake as risk factor for the occurrence of hip fractures. However, there are other factors that intervene with the effect of calcium on bone quality and hip fractures, in particular vitamin D, which plays a crucial role in calcium absorption [51]. It has been shown that there was not much difference between calcium supplementation alone (almost the DRI) or calcium combined with vitamin D on reducing osteoporotic fractures [50, 53]. This is in line with the conditions of use as determined by the European Food Safety Authority that indicate 1,200 mg of calcium per day, or 1,200 mg of calcium and 20 μg of vitamin D per day for women aged 50 years and older (http://www.efsa.europa.eu/). However, if dietary calcium is a threshold nutrient, then that threshold for optimal calcium absorption may be achieved at a lower calcium intake when vitamin D levels are adequate [51]. In this respect, it should be mentioned that the occurrence of dairy food fortification with vitamin D might have been of some influence on the results of our model. However, accurate information on the consumption of such products was not readily available. Besides such a fortification, dairy products themselves contain additional nutrients that are beneficial to bone health, e.g. high protein content [54]. Unfortunately, the literature does not provide valid risk-estimates for osteoporotic fractures given the additional elements in dairy foods. In this regard, the results of this study might give an underestimation about the effect size of dairy calcium. Moreover, other factors mediate the effect of calcium on bone health, and concomitantly on osteoporotic fractures. These factors include exposure to sunlight, level of exercise, and genetic predisposition [55]. Considering the foregoing, it may be expected that there are differences in the relative risk of hip fractures between the populations of different countries.

Our analysis concentrated on the effects of dairy calcium on hip fractures. Two observations need to be made about this. First, we did not include osteoporotic fractures other than hip fractures, due to the unavailability of sufficient data. As a result, our model may have underestimated the beneficial effects of dairy calcium. On the other hand, a side effect of consuming more dairy products might be the intake of more saturated fat, considered a risk factor for vascular diseases. Although dairy products make a contribution to total fat consumption, this contribution is likely to be relatively small. Moreover, a review by Elwood et al. [5] showed that there was no convincing evidence of any increased risk of ischaemic heart disease or ischaemic stroke in subjects who have the highest milk consumption.

For all countries in this study, the loss in quality of life following a hip fracture was based on data from a Swedish study [38] because country-specific data were not available. This should not be considered too much of a limitation, as the quality of life impact of hip fractures is not expected to differ much between countries—not as much as costs might do. Other ‘international’ health-economic studies in the field of osteoporosis followed a similar approach: in these studies, the effect of fractures on quality of life was not based on country-specific sources; whereas for the costs, country-specific data were available [56–59].

## Conclusions

Our study shows that, especially for France and Sweden, the societal burden of hip fractures associated with low calcium intake is quite substantial. Improving the dairy consumption is likely to be effective in decreasing this public health burden and the associated health care expenditures. Our findings support the use of a food-based approach to help maintain bone health or prevent age-related bone loss. This is in line with the position of the French Agency for the Safety of Health Products (AFSSAPS) which recommends to correct calcium and/or vitamin D deficiencies before prescribing anti-osteoporotic drugs [60]. It would be worth performing a cost-effectiveness analysis of a community-based educational health campaign. Behavioral changes, especially related to diet and exercise, form the backbone of public health recommendations for the prevention and treatment of osteoporosis [61], are supported by several RCTs [62, 63] and meta-analyses [50, 64, 65]. Yet, the cost-effectiveness of such recommendations remains largely unexplored.

Our model had to rely on the existing figures that do not take into account the long-term advantages of prevention, mainly focusing on the senior population where bone density is already affected and where dietary interventions will complete the clinical management of diagnosed osteoporosis [66]. Yet, it is no less important to focus on younger people as well, because eating practices established in childhood are likely to be maintained throughout life, and an adequate calcium intake during childhood and adolescence, necessary for the development of peak bone mass, may contribute to bone strength and reduce the risk of osteoporosis and fractures later in life [67, 68].

Although the methods may be further refined, this model appears to be a solid and straightforward, easy-to-use method to assess the health, well-being and cost outcomes of food products from a health economics perspective.
